# Association between Dyslipidemia and Glycated Hemoglobin in a Population-Based Study

**DOI:** 10.3390/metabo14020092

**Published:** 2024-01-26

**Authors:** Purum Kang, Ka Young Kim, Hye Young Shin

**Affiliations:** 1 College of Nursing, Woosuk University, Wanju 55338, Republic of Korea; kpurum@woosuk.ac.kr; 2 Department of Nursing, College of Nursing, Gachon University, Incheon 21936, Republic of Korea; 3 Department of Nursing, Gangseo University, Seoul 07661, Republic of Korea

**Keywords:** cardiovascular disease, diabetes mellitus, dyslipidemia, glycated hemoglobin, HbA1c

## Abstract

Diabetes mellitus and dyslipidemia are well-known risk factors for cardiovascular disease. Unfortunately, the prevalence of dyslipidemia and diabetes mellitus among individuals over 30 years of age in Korea has continuously increased. The current study therefore investigated the association between dyslipidemia and high glycated hemoglobin (Hemoglobin A1c, HbA1c) levels according to age group in adults over 20 years old. We used data from the 7th Korea National Health and Nutrition Examination Survey conducted by the Korea Centers for Disease Control and Prevention from 2016 to 2017. Glycated hemoglobin, a well-established marker for elevated glucose levels, was categorized into three groups, normal (<5.7%), prediabetes (5.7–6.4%), and diabetes (≥6.5%). The presence of dyslipidemia was defined based on a diagnosis of dyslipidemia by a physician. Logistic regression analyses were performed to evaluate the association between the prevalence of dyslipidemia and glycated hemoglobin according to age group. After adjusting for possible confounders, including age, sex, body mass index, marital status, education, occupation, household income, drinking, and smoking, we found a significant increase in the odds ratios (ORs) for dyslipidemia in the prediabetes (OR; 1.915, 95% CI; 1.696 to 2.163) and diabetes (OR; 3.533, 95% CI; 3.019 to 4.134) groups. Among subjects with higher glycated hemoglobin levels, those in their 40s or over had significantly increased odds for dyslipidemia. The current study found an association between high glycated hemoglobin levels and a diagnosis of dyslipidemia among Korean adults. Markers of lipid metabolism in adults with high glycated hemoglobin levels may need to be monitored, especially those in their 40s and older.

## 1. Introduction

Diabetes mellitus and dyslipidemia are well-known risk factors for cardiovascular disease (CVD). Studies have shown that individuals with diabetes mellitus have significantly higher CVD mortality rates compared to those without the diseases [1,2,3] and that the prevalence of dyslipidemia in patients with type 2 diabetes mellitus (T2DM) was significantly high [2,4]. The mechanisms of vascular complication related to diabetes mellitus and dyslipidemia has not been fully explained; in general, however, such mechanisms could be associated with insulin resistance resulting in diabetic dyslipidemia, which involves quantitative and qualitative changes in lipoproteins [2]. Diabetic dyslipidemia includes hypertriglyceridemia, decreased high-density lipoprotein (HDL) cholesterol, and increased small, dense low-density lipoprotein (LDL) cholesterol [2].

In Korea, the prevalence of dyslipidemia and diabetes mellitus among individuals aged over 30 years has been continuously increasing [5,6]. Moreover, a nationwide survey throughout Korea showed that dyslipidemia is more than five times more prevalent among those with T2DM than among those without T2DM (49.5%, 9.7%, respectively) [5]. Furthermore, heart disease ranked third in terms of mortality, with 31 men and 26 women per 100,000 individuals dying from CVD in 2016, values that have consistently increased since 1983 [2,7]. Meanwhile, dyslipidemia and diabetes mellitus have often been prevalent among middle-aged and elderly populations [4,5,6,8], with a previous study reporting high rates of dyslipidemia among individuals with T2DM over 75 years of age [4]. Moreover, evidence has suggested that even younger people with T2DM in their 20s or 30s have at least one lipid parameter that was high [9]. Estimates have shown that the risk of diabetic dyslipidemia with age was perhaps associated with dietary changes based on Western dietary patterns and low physical activity [10]. Thus, tailored interventions focusing on dietary changes and exercise should be provided at an appropriate time for a particular age group to control dyslipidemia and hyperglycemia in the management of diabetes mellitus.

The glycated hemoglobin (Hemoglobin A1c, HbA1c) test evaluates the average level of blood sugar over the past 2 to 3 months and serves as a biomarker to diagnose diabetes [11]. In general, several countries including Korea present diagnostic criteria as normal (<5.7%), prediabetes (5.7–6.4%), and diabetes (≥6.5%), depending on the level of HbA1c [12,13,14]. Moreover, the Korean Diabetes Association recently recommended that fasting plasma glucose or HbA1c be tested annually in case of fasting plasma glucose 100–109 mg/dL or HbA1c level of 5.7–6.0% in the screening criteria and testing methods for high-risk diabetes groups [14].

In this regard, the current study investigated the association between dyslipidemia and diabetes mellitus according to age group in adults over 20 years old using the national population-based survey.

## 2. Materials and Methods

### 2.1. Study Subjects

We used data from the 7th Korea National Health and Nutrition Examination Survey (KNHANES) conducted by the Korea Disease Control and Prevention Agency (KDCA) from 2016 to 2017. The KNHANES was designed as a complex, stratified, multistage, and probability-cluster sampling approach to assess a nationwide population-based survey of the health and nutritional status of Koreans. Among the 16,277 subjects, 11,649 aged over 20 years were included after excluding 4628 who had missing data regarding the diagnosis of diabetes and dyslipidemia. All survey protocols were approved by the Institutional Review Board of the KDCA, and all volunteers provided written informed consent prior to their participation. The study was approved by the Gachon University’s Institutional Review Board (No. 1044396-202208-HR-166-01).

### 2.2. Variables

The demographic variables of participants included age, sex, marital status, education, occupation, household income, and residential area. Marital status was defined as current marital status (yes, no). Education level was classified as elementary school and below, middle school, high school, and university or above. Type of occupation was divided into five groups: white-collar (WC) workers including managers, professionals, and office workers; pink-collar (PC) workers including service and sales workers; blue-collar (BC) workers including technicians and device and machine operators; agribusiness and low-level (AL) workers including skilled workers in agriculture and fishery and low-level laborers; and unemployed. Household income was divided into four levels: lowest, lower middle, upper middle, and highest. Residential area was categorized into urban (administrative divisions of a city) and rural areas (areas not classified as administrative divisions of a city). Furthermore, health-related variables included drinking, smoking, body mass index (BMI), and several blood test indicators. Drinking and smoking were defined based on experience with drinking and smoking (yes, no). BMI was calculated by dividing the weight of participants in kilograms by the square of their height in meters and was used to subsequently categorize them as normal weight (18.5–25.0 kg/m^2^), overweight (25.0–29.9 kg/m^2^), obese (≥30 kg/m^2^), and underweight (<18.5 kg/m^2^). Glycated hemoglobin (Hemoglobin A1C, HbA1c), a well-established marker for elevated glucose levels, was categorized into three groups: normal (<5.7%), prediabetes (5.7–6.4%), and diabetes (≥6.5%). Fasting blood sugar level (mg/dL), total cholesterol (mg/dL), triglycerides (mg/dL), high-density lipoprotein cholesterol (HDL-C, mg/dL), and low-density lipoprotein cholesterol (LDL-C, mg/dL) were used in this study. Moreover, current dyslipidemia treatment status was classified according to whether dyslipidemia was currently treated. The presence of diabetes and dyslipidemia was defined based on having been diagnosed with dyslipidemia by a physician. The survey was performed using interviews conducted by trained investigators and a self-administrated questionnaire.

### 2.3. Statistical Analysis

All analyses were performed using SPSS version 25.0 (IBM Corp., Armonk, NY, USA). Descriptive statistics were applied for all measures. Values are presented as mean ± standard error (SE) or frequencies (%). Chi-square and analysis of variance were used to compare differences among groups. Logistic regression analyses were performed to evaluate the association between the prevalence of dyslipidemia and HbA1c. A *p* value of <0.05 was used to indicate statistical significance.

## 3. Results

### 3.1. General Characteristics of the Study Participants according to the Presence of Dyslipidemia Diagnosis

Table 1 demonstrates the general characteristic of the unweighted and weighted population according to dyslipidemia diagnosis. All variables were presented as unweighted numbers. Categorical variables including sex, marital status, education, occupation, household income, residential area, drinking, smoking, BMI, the presence of diabetes, and current dyslipidemia treatment status were presented as a weighted percentage and continuous variables including age, HbA1c, fasting blood sugar, total cholesterol, triglycerides, HDL-C, and LDL-C were expressed as a weighted mean with standard error. Among the 11,649 subjects included, 2080 were diagnosed with dyslipidemia, and 9569 were not. The mean weighted age was 59.05 years in the group diagnosed with dyslipidemia and 45.13 years in the group without dyslipidemia. Moreover, 53.84% of the participants with dyslipidemia were female, and 46.16% were male, whereas 51.06% of the participants not diagnosed with dyslipidemia were male, and 48.94% were female. Among participants with dyslipidemia, 94.16% were married, and 5.84% were not. Regarding education, 30.68% had an elementary school education or below, 27.02% had a university education or above, 26.68% had a high school education, and 15.62% had a middle school education. Furthermore, 45.10% were unemployed, 16.14% were white-collar workers, 14.92% were low-level workers, 12.10% were pink-collar workers, and 11.73% were blue-collar workers. Regarding household income, 26.46% had the highest household income, 25.51% were upper middle, 24.07% were lower middle, and 23.97% had the lowest household income. For the residential area, 83.57% lived in an urban area, and 16.43% lived in a rural area. Regarding drinking and smoking, 85.14% consumed alcohol, 14.86% did not, 57.98% did not smoke, and 42.02% smoked. In relation to BMI, 50.90% had a normal BMI, 40.82% were overweight, 7.62% were obese, and 0.66% were underweight. Moreover, 74.35% had no diabetes, and 25.65% had diabetes. The mean weighted HbA1c (%) was 6.09 and 5.56 in groups with dyslipidemia and without dyslipidemia, respectively. The mean weighted fasting blood sugar (mg/dL) was 110.57 and 98.34 in the groups with and without dyslipidemia, respectively. The mean weighted total cholesterol (mg/dL), triglycerides (mg/dL), HDL-C (mg/dL), and LDL-C (mg/dL) in the group with dyslipidemia were 188.28, 168.63, 48.88, and 111.79, respectively. Furthermore, the mean weighted total cholesterol, triglycerides, HDL-C, and LDL-C in the group without dyslipidemia were 194.46, 135.81, 51.49, and 121.36, respectively. Among participants with dyslipidemia, 64.07% responded that they were currently receiving treatment, and 35.93% were not.

### 3.2. Characteristics of Participants According to Diagnostic Classifications for Diabetes

Table 2 demonstrates the general characteristics of the study participants according to their HbA1c level, an indicator of diabetes. Among the 11,649 participants included, 7219 had HbA1c levels <5.7% (HbA1c < 5.7%) indicating normal levels, 3274 had HbA1c levels ranging from 5.7% to 6.4% (HbA1c 5.7–6.4%) indicating prediabetes, and 1156 had HbA1c levels of 6.5% or higher (HbA1c ≥ 6.5%) indicating diabetes. Factors including age, sex, marital status, education, occupation, household income, residential area, drinking, smoking, BMI, and dyslipidemia diagnosis were used in this study. The average age was 42.47, 56.00, and 60.07 in the HbA1c < 5.7%, HbA1c 5.7–6.4%, and HbA1c ≥ 6.5% groups, respectively. The majority of the participants in the HbA1c < 5.7% group were females, whereas the majority of those in the HbA1c 5.7–6.4% and HbA1c ≥ 6.5% groups were males. In all groups, there were more married than unmarried participants. Regarding education level, the majority of the participants in the HbA1c < 5.7% and HbA1c 5.7–6.4% groups were university graduates or higher, whereas the majority of those in the HbA1c ≥ 6.5% group reached elementary school or below. Regarding occupation, the largest proportion in all groups was unemployed. The majority of the participants in the HbA1c < 5.7% and HbA1c 5.7–6.4% groups had the highest household income level, whereas the majority of those in the HbA1c ≥ 6.5% group had the lowest household income level. Moreover, all groups including HbA1c < 5.7%, HbA1c 5.7–6.4%, and HbA1c ≥ 6.5% showed higher percentages of urban residence, drinking alcohol, non-smoking, normal BMI, and the absence of a dyslipidemia diagnosis.

### 3.3. Odds Ratio for Dyslipidemia according to Age Group and HbA1c Levels

Logistic regression analyses were performed to estimate the odds of dyslipidemia according to age group and HbA1c levels (Table 3). In total, significantly higher ORs were observed in the HbA1c 5.7–6.4% (OR; 1.932, 95% CI; 1.716 to 2.175) and HbA1c ≥ 6.5% (OR; 3.446, 95% CI; 2.959 to 4.012) groups after adjusting for age, sex, and BMI (Model 1). Further adjustments for possible confounders, including age, sex, BMI, marital status, education, occupation, household income, drinking, and smoking (Model 2), also showed significantly higher ORs in the HbA1c 5.7–6.4% (OR; 1.915, 95% CI; 1.696 to 2.163) and HbA1c ≥ 6.5% (OR; 3.533, 95% CI; 3.019 to 4.134) groups. Among the participants in the HbA1c 5.7–6.4% group, those in their 30s showed significantly higher ORs in Models 1 and 2, and HbA1c ≥ 6.5% groups showed significantly higher ORs in Model 1. Among the participants in the HbA1c 5.7–6.4% and HbA1c ≥ 6.5% groups, those in their 40s (OR; 1.877, 7.217), 50s (OR; 1.973, 4.381), 60s (OR; 1.609, 2.627), and 70s or above (OR; 1.395, 2.126) had significantly higher odds for dyslipidemia under Model 1. Furthermore, among the participants in HbA1c 5.7–6.4% and HbA1c ≥ 6.5% groups, those in their 40s (OR; 1.786, 6.787), 50s (OR; 1.994, 4.528), 60s (OR; 1.558, 2.740), and 70s or above (OR; 1.378, 2.237) had significantly higher odds for dyslipidemia under Model 2.

## 4. Discussion

Our results showed that high HbA1c levels were associated with a diagnosis of dyslipidemia, independent of age, sex, BMI, marital status, education, occupation, household income, and other health related habits. Moreover, the present study also found that the association between diabetes status and dyslipidemia was prominent among middle-aged subjects, especially those in their 40s and older.

Our study confirmed the relationship between high HbA1c levels and dyslipidemia using cross-sectional data. The high HbA1c value does not simply mean that the patient is diabetic, but that the blood glucose is not properly regulated. Lipid and glucose metabolism are simultaneously controlled by complex mechanisms at the molecular level, with insulin resistance and inflammation playing a crucial role [15,16]. Insulin not only modulates glucose dynamics but also suppresses very low-density lipoprotein secretion and promotes degradation of triglyceride-rich lipoproteins [17]. Previous studies have reported that abnormal lipid profiles in obese children were associated with insulin resistance [18], and a cross-sectional study revealed that subclinical inflammation was associated with impaired fasting glucose and abnormal lipid levels among Korean adults [19]. And also, high-sensitivity C-reactive protein is reported to be related to cholesterol levels and HbA1c in Korean adolescents [20]. Therefore, although this cross-sectional study could not definitively determine the mechanisms involved, our findings suggested that dyslipidemia was associated with inappropriate blood glucose metabolism and was likely associated with these molecular mechanisms such as inflammation and insulin resistance. Therefore, it is necessary for people with poor sugar control to monitor lipid-related indicators. In Korea, blood glucose is required to be tested every two years for general adult health examinations, whereas lipid profiles are evaluated only once every four years for women over 40 years old and men over 24 years old [21]. Therefore, it is necessary to recommend not only lifestyle education for glucose control but also the management of lipid levels in subjects with inadequate blood glucose control.

In this study, it was confirmed that a high HbA1c level and dyslipidemia are related even after correction for many lifestyle-related variables such as drinking and occupation. Previous studies have also shown that cholesterol levels are associated with prediabetes status, even after adjusting for age or body mass index [22]. However, this study did not account for all lifestyle factors, such as nutrition habits. Serum glucose and lipid metabolism control are affected by lifestyle that causes metabolic abnormalities including dietary factors, and a previous literature review reported that lifestyle intervention could adjust both glucose and lipid levels [23]. Indeed, many people have dyslipidemia and type 2 diabetes mellitus simultaneously, which are types of metabolic abnormalities [24]. Previous studies have shown that the higher the fasting blood glucose level in type 2 diabetic patients, the higher the blood cholesterol level [25]. Thus, lifestyle including dietary factors cannot be completely excluded, and it would be a “bridge” of association between high HbA1c and dyslipidemia.

Above all, dyslipidemia and HbA1c to reflect glucose control homeostasis were not statistically significant in the subjects in their 20s and only subjects with a HbA1c level at the prediabetic level in their 30s in this study. However, the HbA1c level and diagnosis of dyslipidemia were statistically significant those in their 40s and older. In this study, different results according to the age group may be due to differences in the reasons for inadequate blood glucose control by age group. This is presumed to be due to T1DM being the mainstream in the under 30s, which is caused by innate insulin deficiency rather than problems related to metabolism in the body [26]. Type 1 diabetes is caused by an autoimmune disease characterized by the destruction of beta cells in the pancreas and has less environmental impact [27]. On the other hand, Type 2 diabetes appears in relatively older people and results from increased insulin resistance with relative insulin insufficiency, which is related to environmental factors such as lifestyle [26]. Therefore, the association between glucose homeostasis and a diagnosis of dyslipidemia in this study is more likely to be related to relative insulin deficiency than to innate insulin deficiency. Previous studies have shown that people with type 2 diabetes who have high HbA1c levels have higher blood lipid levels than those who do not [28].

In particular, our results showed that the relationship between HbA1c levels and the diagnosis of dyslipidemia was most pronounced in individuals aged 40 and older, thus careful management is especially crucial for this age group. And by tracing the trend in lipid profiles in Korean Adults over the past decade, the prevalence of hypercholesterolemia significantly increased in those in their 40s and older [29]. In addition, descriptive studies conducted in Nepal also showed that most of the patients with HbA1c-based prediabetes were between 45 and 64 years old [30]. Therefore, it is particularly necessary to strongly recommend the lipid profile test and simultaneous management for middle-aged individuals with inadequately managed blood glucose because compared to lipid profiles, blood sugar is relatively easy to measure and accessible. And also, lipid profiles are affected by recent intake, whereas HbA1c is a long-term marker for blood glucose control with minimal considerable impact on glucose level such as recent intake [31]. Therefore, considering the high prevalence of glucose and lipid metabolism disorders and their importance as predictors of cardiovascular diseases, it is considered meaningful to continuously monitor the lipid profile in subjects with inadequate glucose control and normal lipid levels.

### Strengths and Limitations

This study presents several strengths and limitations. Our study conducted an analysis of a large cross-sectional dataset, and these data are representative of the Korean population. Thus, we were able to provide important insights into the nature of the association between a high HbA1c level and the diagnosis of dyslipidemia considering the age groups in Korea. Despite these strengths, one of the limitations of this cross-sectional analysis is that it cannot deduce causation. And the dyslipidemia variable in this study was based on the diagnosis by a doctor, not the actual prevalence. However, since the variable of being diagnosed by a doctor has applied stricter criteria, we propose a future study that uses the actual concentration of the lipid profile as a variable to compare the results. Finally, since this study was conducted in Korea which has single race and cultural characteristics, there may be differences depending on culture and race, thus further research is needed on other cultures or races.

## 5. Conclusions

In conclusion, there is an association between a high HbA1c level and the diagnosis of dyslipidemia in Korean adults. Marker of lipid metabolism including LDL, HLD, and total cholesterol triglyceride in adults with high HbA1c may need to be monitored, especially for those in their 40s and older. Prospective and biochemical research is necessary to clarify the role of glucose homeostasis in middle-aged adults with dyslipidemia.

## Figures and Tables

**Table 1:** General characteristics of the study participants according to dyslipidemia diagnosis (n = 11649).

Characteristics		Dyslipidemia				*p* Value
		Yes		No		
		Unweighted No.	Weighted % (SE)	Unweighted No.	Weighted % (SE)	
Age (years) ^+^		2080	59.05 (0.36)	9569	45.13 (0.28)	<0.001
Sex	Male	817	46.16 (1.27)	4334	51.06 (0.53)	0.001
	Female	1263	53.84 (1.27)	5235	48.94 (0.53)	
Marital status	Yes	2001	94.16 (0.77)	7847	75.00 (0.76)	<0.001
	No	79	5.84 (0.77)	1722	25.00 (0.76)	
Education	≤Elementary school	737	30.68 (1.31)	1604	12.01 (0.53)	<0.001
	Middle school	328	15.62 (0.95)	804	7.73 (0.36)	
	High school	503	26.68 (1.17)	2898	35.12 (0.80)	
	≥University	432	27.02 (1.51)	3761	45.13 (1.02)	
Occupation	WC worker	258	16.14 (1.12)	2514	30.74 (0.79)	<0.001
	PC worker	221	12.10 (0.88)	1125	13.05 (0.48)	
	BC worker	182	11.73 (0.92)	958	12.40 (0.51)	
	AL worker	332	14.92 (1.00)	1123	10.24 (0.50)	
	unemployed	1008	45.10 (1.33)	3343	33.57 (0.69)	
Household income	Lowest	578	23.97 (1.31)	1654	14.10 (0.70)	<0.001
	Lower middle	549	24.07 (1.18)	2263	22.89 (0.71)	
	Upper middle	480	25.51 (1.30)	2714	30.43 (0.82)	
	Highest	462	26.46 (1.50)	2906	32.58 (1.11)	
Residential area	Urban	1667	83.57 (1.87)	7798	84.82 (1.71)	0.255
	Rural	413	16.43 (1.87)	1771	15.18 (1.71)	
Drinking	No	353	14.86 (0.99)	952	7.85 (0.35)	<0.001
	Yes	1709	85.14 (0.99)	8497	92.15 (0.35)	
Smoking	No	1296	57.98 (1.28)	5602	55.89 (0.64)	0.163
	Yes	765	42.02 (1.28)	3837	44.11 (0.64)	
BMI	Normal	1079	50.90 (1.25)	6018	62.42 (0.60)	<0.001
	Overweight	831	40.82 (1.17)	2653	27.95 (0.56)	
	Obesity	156	7.62 (0.70)	460	5.07 (0.28)	
	Underweight	13	0.66 (0.20)	423	4.56 (0.26)	
Diabetes	No	1524	74.35 (1.15)	8977	95.40 (0.27)	<0.001
	Yes	556	25.65 (1.15)	592	4.60 (0.3)	
HbA1c ^+^		2080	6.09 (0.03)	9569	5.56 (0.01)	<0.001
Fasting blood sugar ^+^		2080	110.57 (0.89)	9569	98.34 (0.30)	<0.001
Total cholesterol ^+^		2080	188.28 (1.17)	9569	194.46 (0.49)	<0.001
Triglycerides ^+^		2080	168.63 (4.49)	9569	135.81 (1.78)	<0.001
HDL-C ^+^		2080	48.88 (0.31)	9569	51.49 (0.17)	<0.001
LDL-C ^+^		461	111.79 (2.12)	1394	121.36 (1.08)	<0.001
Current dyslipidemia treatment status	No	672	35.93 (1.27)			<0.001
	Yes	1408	64.07 (1.27)			

WC: white-collar; PC: pink-collar; BC: blue-collar; AL: agribusiness and low-leveled; BMI: body mass index; HDL-C: high-density lipoprotein-cholesterol; LDL-C: low-density lipoprotein-cholesterol; SE: standard error; ^+^ weighted mean with SE.

**Table 2:** Characteristics of participants according to HbA1c level.

Characteristics		HbA1c (Weighted % (SE))			*p* Value
		HbA1c < 5.7 (*n* = 7219)	5.7 ≤ HbA1c < 6.5 (*n* = 3274)	HbA1c ≥ 6.5 (*n* = 1156)	
Age (years) ^+^		42.47 (0.28)	56.00 (0.34)	60.07 (0.52)	<0.001
Sex	Male	49.81 (0.63)	50.25 (0.92)	54.57 (1.61)	0.033
	Female	50.19 (0.63)	49.75 (0.92)	45.43 (1.61)	
Marital status	Yes	70.51 (0.90)	92.22 (0.64)	95.58 (0.81)	<0.001
	No	29.49 (0.90)	7.78 (0.64)	4.42 (0.81)	
Education	≤Elementary school	9.36 (0.48)	24.14 (1.03)	32.52 (1.77)	<0.001
	Middle school	6.62 (0.38)	12.84 (0.75)	16.32 (1.26)	
	High school	35.18 (0.86)	30.91 (1.13)	31.43 (1.80)	
	≥University	48.85 (1.06)	32.11 (1.36)	19.73 (1.62)	
Occupation	WC worker	33.14 (0.87)	20.89 (0.98)	13.13 (1.26)	<0.001
	PC worker	12.96 (0.53)	12.86 (0.72)	12.53 (1.33)	
	BC worker	11.30 (0.55)	13.68 (0.82)	16.31 (1.51)	
	AL worker	9.01 (0.47)	15.01(0.89)	14.78 (1.48)	
	unemployed	33.59 (0.75)	37.55 (1.07)	43.24 (1.79)	
Household income	Lowest	11.80 (0.67)	21.55 (1.09)	28.90 (1.63)	<0.001
	Lower middle	22.46 (0.79)	24.57 (1.02)	23.59 (1.51)	
	Upper middle	31.58 (0.90)	25.90 (1.01)	25.54 (1.49)	
	Highest	34.17 (1.18)	27.99 (1.26)	21.97 (1.54)	
Residential area	Urban	86.36 (1.61)	81.56 (2.06)	79.71 (2.40)	<0.001
	Rural	13.64 (1.61)	18.44 (2.06)	20.29 (2.40)	
Drinking	No	6.41 (0.33)	12.84 (0.68)	17.82 (1.33)	<0.001
	Yes	93.59 (0.33)	87.16 (0.68)	82.18 (1.33)	
Smoking	No	57.77 (0.72)	53.73 (1.02)	50.84 (1.75)	<0.001
	Yes	42.23 (0.72)	46.27 (1.02)	49.16 (1.75)	
BMI	Normal	66.16 (0.67)	51.22 (1.05)	43.98 (1.63)	<0.001
	Overweight	25.14 (0.65)	38.99 (1.02)	41.79 (1.56)	
	Obesity	3.64 (0.27)	7.62 (0.56)	13.81 (1.16)	
	Underweight	5.06 (0.31)	2.17 (0.28)	0.42 (0.20)	
Dyslipidemia	No	91.59 (0.38)	74.87 (0.90)	58.19 (1.87)	<0.001
	Yes	8.41 (0.38)	25.13 (0.90)	41.81 (1.87)	

WC: white-collar; PC: pink-collar; BC: blue-collar; AL: agribusiness and low-leveled; BMI: body mass index; Normal: HbA1c < 5.7; Prediabetes: 5.7 ≤ HbA1c < 6.5; Diabetes: HbA1c ≥ 6.5; ^+^ weighted mean with SE.

**Table 3:** Odds ratio for dyslipidemia according to age groups.

	Total			Age Groups			
		20s	30s	40s	50s	60s	70+
Model 1							
HbA1c < 5.7							
Ref	1	1	1	1	1	1	1
5.7 ≤ HbA1c < 6.5							
OR	1.932	0.888	2.548	1.877	1.973	1.609	1.395
95% CI	1.716–2.175	0.102–7.721	1.433–4.534	1.319–2.672	1.581–2.462	1.301–1.989	1.099–1.769
*p*-value	<0.001	0.914	0.001	<0.001	<0.001	<0.001	0.006
HbA1c ≥ 6.5							
OR	3.446	3.535	3.207	7.217	4.381	2.627	2.126
95% CI	2.959–4.012	0.311–40.124	1.007–10.218	4.654–11.192	3.226–5.951	2.008–3.436	1.612–2.803
*p*-value	<0.001	0.308	0.049	<0.001	<0.001	<0.001	<0.001
Model 2							
HbA1c<5.7							
Ref	1	1	1	1	1	1	1
5.7 ≤ HbA1c < 6.5							
OR	1.915	2.020	2.812	1.786	1.994	1.558	1.378
95% CI	1.696–2.163	0.223–18.272	1.566–5.049	1.239–2.575	1.588–2.504	1.253–1.936	1.075–1.766
*p*-value	<0.001	0.531	0.001	0.002	<0.001	<0.001	0.011
HbA1c ≥ 6.5							
OR	3.533	11.643	2.329	6.787	4.528	2.740	2.237
95% CI	3.019–4.134	0.723–187.513	0.628–8.631	4.264–10.802	3.298–6.217	2.075–3.619	1.676–2.986
*p*-value	<0.001	0.083	0.206	<0.001	<0.001	<0.001	<0.001

Normal: HbA1c < 5.7; Prediabetes: 5.7 ≤ HbA1c < 6.5; Diabetes: HbA1c ≥ 6.5; OR: odds ratio; 95% CI: 95% confidence interval; Model 1: adjusted for age, sex (ref: male), and BMI (ref: normal); Model 2: adjusted for age, sex (ref: male), BMI (ref: normal), marital status (ref: yes), education (ref: elementary school and below), occupation (ref: white-collar worker), household income (ref: lowest), drinking (ref: no), and smoking (ref: no).

## Data Availability

All data used were from the 7th Korea National Health and Nutrition Examination Survey conducted by the Korea Disease Control and Prevention Agency (https://www.kdca.go.kr/yhs/yhshmpg/result/yhsresult/rawDtaList.do).
